# Neural speech encoding in fetal alcohol spectrum disorder: an exploratory study using frequency-following responses

**DOI:** 10.3389/fnins.2026.1871045

**Published:** 2026-07-16

**Authors:** Sheila Templado, Guillermo Savio, Francisco García-Purriños

**Affiliations:** 1 Catholic University San Antonio of Murcia (UCAM), Murcia, Spain; 2 R&D Department, Intelligent Hearing Systems, Miami, FL, United States; 3 Department of Otolaryngology, Los Arcos del Mar Menor University Hospital, San Javier, Murcia, Spain

**Keywords:** auditory processing, FASD, fetal alcohol spectrum disorders, FFR, frequency-following responses

## Abstract

**Background:**

Fetal alcohol spectrum disorder (FASD) is associated with neurodevelopmental impairments, including listening difficulties not always explained by peripheral hearing loss, suggesting alterations at the level of neural auditory processing. The frequency-following response (FFR) provides an objective measure of neural speech encoding and may offer insight into auditory function in this population.

**Methods:**

Twenty-five normal-hearing participants were included: 11 individuals with FASD and 14 controls. Speech-evoked FFRs were recorded using a 160 ms /da/ stimulus at 80 dB SPL with a stimulation rate of 4.35/s. Pitch tracking, stimulus–response correlation, response latency, and signal quality were analyzed using non-parametric statistics. Given the exploratory nature of the study and uncontrolled demographic variables — including a significant age difference between groups — findings should be interpreted as preliminary and hypothesis-generating.

**Results:**

Compared to controls, individuals with FASD showed reduced pitch-tracking consistency and lower stimulus–response correlation, with the most robust finding being a large-effect reduction in F0-range correlation (R[70–120 Hz]: *p* < 0.001, rank-biserial *r* = 0.823). Prolonged latencies were observed across multiple response components, and signal-to-noise ratio tended to be lower in the FASD group.

**Conclusion:**

This exploratory proof-of-concept study provides preliminary evidence of altered neural speech encoding in individuals with FASD despite normal peripheral hearing. Given uncontrolled confounds, observed differences cannot be specifically attributed to FASD. These findings establish the feasibility of FFR in this population and provide the empirical foundation for future controlled research on auditory biomarkers and intervention monitoring in FASD.

## 1 Introduction

Fetal Alcohol Spectrum Disorder (FASD) is a diagnostic term encompassing the teratogenic effects of prenatal alcohol exposure. These preventable alterations result from maternal alcohol consumption during pregnancy. FASD is currently considered a major public health concern and represents a leading cause of acquired intellectual disability in the United States. Importantly, it is a condition that can be identified, prevented, and avoided.

Globally, the prevalence of alcohol consumption during pregnancy is estimated at approximately 10%, with the highest rates reported in Ireland (over 60%) and the United Kingdom (up to 49%). Russia and Australia follow with rates between 30% and 39%, while prevalence in European and North American countries is approximately 25% (Popova et al., 2017).

FASD comprises a spectrum of conditions that vary according to phenotypic expression, including fetal alcohol syndrome (FAS), partial fetal alcohol syndrome (pFAS), alcohol-related birth defects (ARBD), and alcohol-related neurodevelopmental disorder (ARND). This heterogeneity reflects the timing, duration, and magnitude of prenatal alcohol exposure, as well as its effects on placental permeability and fetal development.

Exposure during the first trimester is strongly associated with congenital malformations, as this period corresponds to organogenesis. However, cognitive and behavioral consequences extend beyond early gestation and may also arise from exposure during the second and third trimesters, reflecting the continuous development of the central nervous system throughout pregnancy. For these reasons, no safe level of prenatal alcohol exposure has been established (Wozniak et al., 2019; Evrard, 2010; Lipinski et al., 2012; Coles, 1994).

Individuals with FASD are typically assessed within the fields of psychology and psychiatry, and most of the available literature—including studies related to hearing—has been developed within these domains. However, emerging evidence suggests that auditory difficulties in this population may not be fully explained by peripheral hearing loss.

McLaughlin and colleagues, in a study involving more than 300 children, reported that although the prevalence of sensorineural or conductive hearing loss was higher in children with FAS, this was not the case in individuals without congenital malformations or dysmorphia within the spectrum, where hearing thresholds were comparable to those of typically developing peers. Importantly, the study highlighted that listening difficulties in the absence of hearing loss were highly prevalent and could significantly impact language development, social interaction, and cognitive performance (McLaughlin et al., 2019). Auditory function in individuals with FASD has traditionally been assessed using behavioral measures, which require active participation and sustained attention. However, these requirements may limit the reliability of such assessments in this population, given the frequent presence of attentional and behavioral difficulties (Felgueras et al., 2024).

Objective electrophysiological techniques provide an alternative approach to the study of auditory function, allowing the evaluation of neural processing independently of behavioral performance. Previous studies using magnetoencephalography have reported delays in auditory processing in children with FASD (Crichton et al., 2024; Stephen et al., 2012), while quantitative electroencephalography (QEEG) has revealed alterations in interhemispheric connectivity (Gerstner et al., 2024; Mattson et al., 1992). In addition, event-related potentials (ERPs) have shown deficits in auditory processing and attentional mechanisms in this population. Specifically, Gerhold et al. (2017) reported reduced P2 amplitude, increased N2 amplitude, and prolonged P3 latency with reduced amplitude in children with FASD during an auditory Go/NoGo task, suggesting impaired stimulus discrimination, conflict monitoring, and attentional allocation. Lane et al. (2014) further demonstrated complexities in attentional functioning in FASD, with parental reports of attention problems not consistently aligned with standardized performance measures, highlighting the heterogeneity of attentional deficits in this population. However, these approaches may still be influenced by task demands or require a certain degree of cooperation.

The American Speech-Language-Hearing Association (ASHA), supported by the American Academy of Audiology (AAA), defines auditory processing disorder (APD) as a deficit in the perceptual processing of auditory information within the central nervous system, and emphasizes that the underlying neurobiological activity can be assessed through auditory evoked potentials (Musiek and Chermak, 2013; Schow et al., 2000).

To investigate these auditory processing differences from a neurophysiological perspective, the Frequency-Following Response (FFR) was selected as the primary measure. The FFR reflects the neural encoding of sound, particularly its temporal and spectral features, and provides insight into the fidelity with which acoustic information is represented within the auditory system.

This response is characterized by phase-locked neural activity that closely follows the periodic properties of the stimulus, allowing the evaluation of key aspects of speech processing such as pitch, timing, and harmonic structure (Kraus, 2011; Kraus et al., 2017; Barman et al., 2022; Coffey et al., 2016; Krizman and Kraus, 2019; Schochat et al., 2017; Kraus, 2021).

Traditionally, FFR has been associated with subcortical generators, particularly within the midbrain, although recent evidence suggests contributions from multiple levels of the auditory system, including cortical sources and corticofugal modulation (Escera, 2023; Gorina-Careta et al., 2021).

In this context and given the presence of listening difficulties in individuals with FASD despite normal peripheral hearing, the present study aims to characterize neural speech encoding using FFR in this population, and to compare these responses with those obtained in a group of typically developing individuals with normal hearing.

## 2 Materials and methods

A quasi-experimental descriptive design was employed to compare auditory responses between individuals with FASD and a control group. Inclusion criteria for the FASD group consisted of a confirmed medical diagnosis of FASD and no evidence of peripheral hearing loss. One participant was excluded due to excessive hyperactivity, which prevented the maintenance of the relaxed conditions required for reliable electrophysiological recording.

For the control group, inclusion criteria included normal peripheral hearing, no history of prenatal exposure to alcohol, and age within the approximate range of the study group. Participants also presented a medium-to-high academic and intellectual level. The study was approved by the Ethics Committee of the International Doctoral School of the Catholic University of Murcia.

### 2.1 Study sample

A total of 25 participants with normal peripheral hearing were included. The FASD group comprised 11 individuals (5 females, 6 males; mean age = 17.14 years, SD = 6.63, range = 7–25 years) with a confirmed diagnosis. The control group included 14 participants (7 females, 7 males; mean age = 26.71 years, SD = 6.50, range = 19–40 years), selected to approximate the age range of the study group. The two groups differed significantly in age (Mann–Whitney U = 25.5, *p* = 0.005, rank-biserial *r* = 0.67), a difference that is explicitly acknowledged as a study limitation.

Participants with FASD were recruited through a local association of families with adopted children diagnosed by the regional medical unit in Murcia, Spain. All presented neurodevelopmental and behavioral difficulties, including reduced speech intelligibility in noisy environments, attentional and memory deficits, and academic challenges. Phenotypic features associated with FASD were present in most cases; however, one participant presented no morphological markers and was diagnosed based on maternal history and clinical neurodevelopmental criteria. One FASD participant was excluded prior to recording due to excessive motor restlessness that prevented electrode placement. No electrophysiological data were collected from this individual.

Control participants were recruited from the Biomedical Engineering Research Laboratory at the University of Miami (USA), the Templado Advanced Audiology and ENT Clinic, and Los Arcos del Mar Menor University Hospital (Murcia, Spain). Inclusion criteria included normal peripheral hearing, no history of prenatal exposure to toxins, and high academic performance.

All participants were native Spanish speakers. Control participants recruited at the University of Miami belonged to the local Hispanic/Latino community, with Spanish as their dominant language; some individuals in this subgroup may also speak English as a second language.

All participants underwent otoscopic examination prior to testing. Ears presenting transient peripheral conditions were excluded from analysis. Specifically, one ear in the control group was excluded due to acute otitis media at the time of examination. Ears with excessive cerumen were cleaned under otomicroscopy by an ENT specialist prior to recording to ensure optimal recording conditions.

FFR recordings were obtained monaurally, and not all control participants contributed recordings from both ears. Only valid ear-level recordings were included in the analysis, resulting in a final dataset of 43 ears (22 FASD, 21 controls). Given that both ears from the same participant may not be statistically independent, this ear-level approach should be considered exploratory.

### 2.2 Materials

Recordings were obtained using the IHS SmartEP® system (IHS, USA). FFR analysis was performed using the Brainstem Toolbox (MATLAB®, Northwestern University, USA). Statistical analyses were conducted using the JAMOVI® software package.

### 2.3 Stimuli

The speech-evoked stimulus used to elicit electrophysiological responses was the syllable /da/, generated using the Advanced Auditory Research Module of the SmartEP® system (IHS, USA). The same digital stimulus file was used across both recording sites (Murcia, Spain and Miami, USA). Recordings were obtained using the IHS SmartEP® system with the same hardware configuration (Duet®) at both sites, ensuring equivalent stimulus delivery and calibration conditions. The /da/ syllable was selected because it is a synthetic, digitally generated speech token with well-characterized acoustic parameters, whose neural correlates have been extensively described in the FFR literature (Russo et al., 2004; Skoe and Kraus, 2010). A 160 ms duration was chosen to provide access to both transient onset components (V, A), the sustained transition component (C), the sustained periodic components (D, E, F) corresponding to the fundamental frequency and its harmonics, and the offset component (O), the latter group being particularly relevant for characterizing temporal synchrony deficits. The stimulus fundamental frequency (F0 ≈ 97.66 Hz) lies within a low-frequency range for which the FFR reflects the integrated activity of subcortical (midbrain and thalamic) and cortical generators (Gorina-Careta et al., 2021).

### 2.4 Procedure

Prior to electrophysiological recording, all participants underwent otoscopy, transient-evoked otoacoustic emissions (TEOAEs), and pure-tone audiometry (PTA), confirming hearing thresholds within normal limits. Participants were instructed to remain relaxed and minimize muscle activity during the recordings. The order of protocol presentation, as well as the ear tested first, was randomized.

Four surface electrodes were placed according to the international 10–20 EEG system, with the active electrode at Cz, the reference electrode at the ipsilateral mastoid (M1 or M2), and the ground electrode (GND) at Fpz.

Electrode impedances were maintained below 2 kΩ at all recording sites. Although the system allows for dual-channel acquisition, recordings were performed separately for each ear using a single-channel configuration corresponding to the stimulated ear.

Insert earphones (ER-3A, Etymotic Research, USA) were used in all cases and calibrated according to the manufacturer’s standard clinical procedures.

Recordings were conducted in a sound-attenuated booth.

### 2.5 Protocol

Frequency-Following Responses (FFRs) were recorded using the IHS SmartEP® system with Duet® hardware (Intelligent Hearing Systems, Miami, FL, USA). Four surface electrodes were positioned according to the International 10–20 EEG system: active electrode at Cz, reference at the ipsilateral mastoid (M1 or M2), and ground at Fpz. Electrode impedances were maintained below 2 kΩ at all recording sites.

The /da/ stimulus (F0 = 97.66 Hz; duration = 160 ms; rate = 4.35/s) was presented monaurally at 80 dB SPL via ER-3 insert earphones (Etymotic Research, USA), calibrated according to the manufacturer’s standard clinical procedure. Stimulus polarity was set to alternating to minimize stimulus artifact and cochlear microphonic contributions. Recordings were conducted inside a sound-attenuated and electrically shielded booth.

Signals were amplified (gain = 100,000), digitized at 13.33 kHz, and recorded using an online band-pass filter of 30–3,000 Hz. A pre-stimulus baseline of 40 ms was applied, with a total recording window of 200 ms. Online artifact rejection was set at ±31 μV. Each stimulus condition consisted of three independent blocks of 1,024 sweeps (3,072 sweeps per ear per session), representing a theoretical stimulus presentation time of approximately 11.8 min per ear.

Accepted sweeps were averaged to obtain one waveform per block; block-level waveforms were retained separately for subsequent analyses.

Offline digital band-pass filtering (70–2,000 Hz) was applied to all block-level waveforms prior to analysis. Pitch tracking was computed using an autocorrelation-based method across 0–150 ms, with a 40-ms sliding window and 1-ms step size, following established FFR analysis procedures. Pitch strength was defined as the normalized autocorrelation coefficient at the lag corresponding to the stimulus F0; pitch error was calculated as the absolute difference between the stimulus F0 and the extracted neural pitch estimate. Latency identification of FFR components (V, A, C, D, E, F, O) was performed manually by a single trained evaluator.

Signal-to-noise ratio (SNR) was extracted automatically by the SmartEP® system from the electrophysiological recording within the 20–140 ms time window, as defined by the system’s default parameters.

### 2.6 Data analysis

Due to the small sample size and the high inter-individual variability observed, non-parametric statistical analyses were applied without assuming normal distribution of the data. Between-group comparisons were performed using the Mann–Whitney U test, with statistical significance set at *p* < 0.05.

Given the exploratory nature of the study and the available sample size, analyses were conducted independently for each variable to identify potential group differences. As the unit of analysis was based on ear-level recordings, results should be interpreted with caution, considering the potential lack of full independence between ears from the same participant.

In addition, data visualization was prioritized, including both group-level summaries and individual data points, to provide a more comprehensive representation of variability and to avoid overinterpretation based solely on statistical significance.

The ear was used as the unit of analysis (*n* = 43 ears: 22 FASD, 21 control). This decision is grounded in the well-established functional asymmetry of auditory pathways during monaural stimulation: FFR responses differ as a function of the stimulated ear, reflecting lateralization of speech encoding at the subcortical level (Hornickel et al., 2009). Each ear therefore constitutes a functionally distinct unit of observation during monaural stimulation. No between-ear comparison (laterality analysis) was performed, as this was outside the scope of the present exploratory study and the sample was not powered for such analysis.

No correction for multiple comparisons was applied. Results with *p*-values close to the significance threshold should be interpreted with appropriate caution and are considered preliminary and hypothesis-generating. The stimulus–response correlation in the F0 range (R[70–120 Hz]) constitutes the most robust finding of the study.

## 3 Results

### 3.1 Participant characteristics

The FASD group comprised 11 individuals (5 females, 6 males), with a mean age of 17.14 years (SD = 6.63; median = 20.0; IQR = 10.5–22.0; range = 7–25). The control group included 14 participants (7 females, 7 males), with a mean age of 26.71 years (SD = 6.50; median = 25.5; IQR = 21.2–29.5; range = 19–40). A Mann–Whitney U test revealed a statistically significant difference in age between groups (U = 25.5, *p* = 0.005, rank-biserial r = 0.67), confirming a substantial age gap. Sex distribution did not differ markedly between groups. These demographic differences are considered in the interpretation of findings and are explicitly addressed in the limitations section.

### 3.2 FFR results

**Figure 1:**
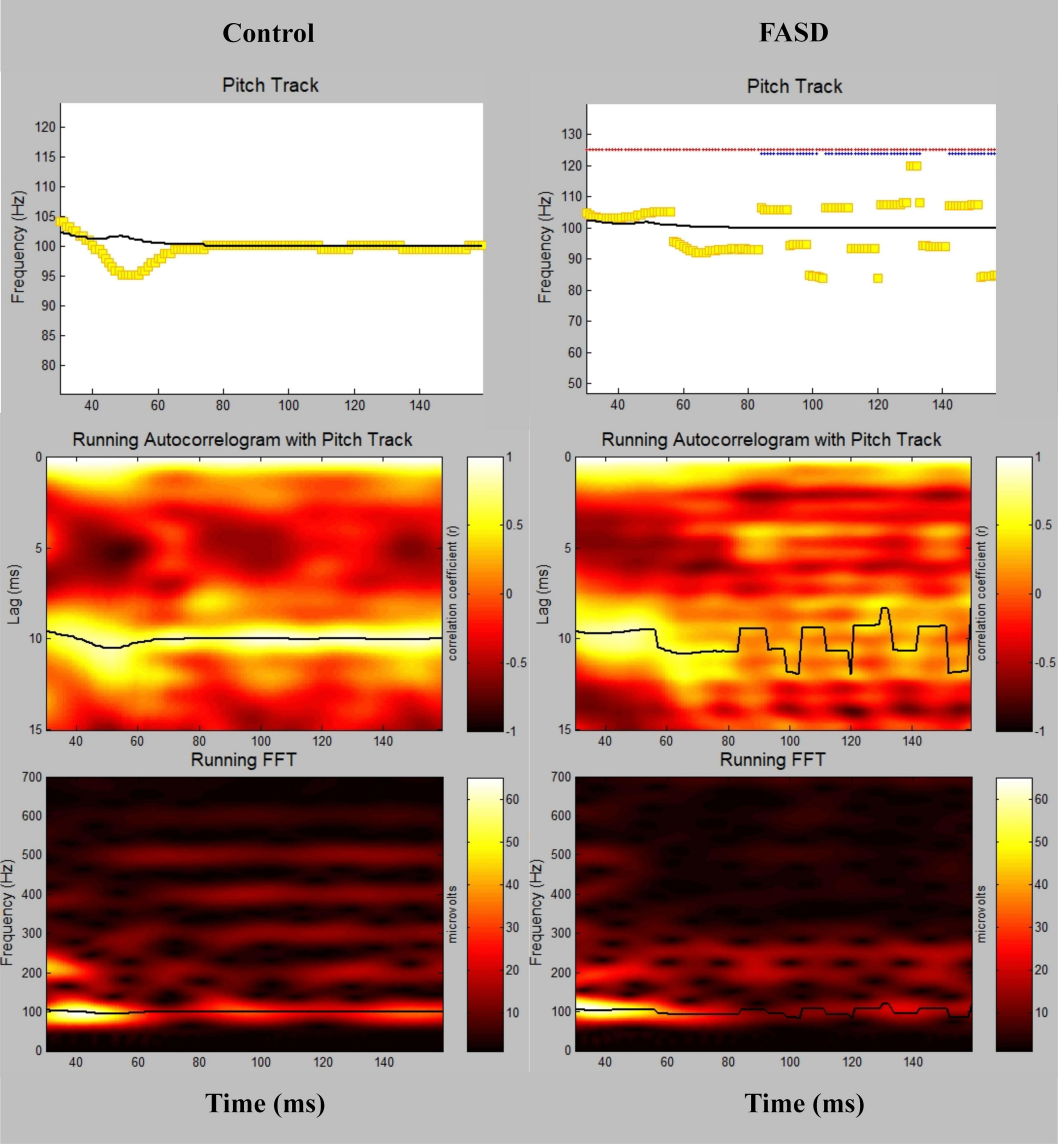
Frequency-domain analysis of the FFR. Representative examples of pitch tracking in a control participant and a participant with FASD. The figure shows an example of frequency-domain analysis of the FFR in a typical control participant (*left panel*) and a typical participant with FASD (*right panel*). The top panels show pitch tracking of the response, where the black line represents the F0 of the stimulus and the yellow points indicate the periodicity of the neural response. The middle panels show the running autocorrelogram of the FFR response, with the superimposed line indicating the temporal tracking of the fundamental periodicity. The bottom panels represent the running fast Fourier transform (FFT), obtained using short-time Fourier transform (STFT), where areas of higher color intensity indicate greater spectral energy.

Representative examples of frequency-domain analysis of FFR are shown in Figure 1. For a control participant and a participant with FASD.

In the top panels, pitch tracking of the response is displayed, where the black line represents the F0 of the stimulus and the yellow markers indicate the periodicity of the neural response. The control participant shows a close correspondence between the stimulus F0 and the neural response, whereas the FASD participant exhibits greater variability in the tracking of the response.

In the middle panels, the running autocorrelogram illustrates the temporal periodicity of the response. A clear and stable periodic structure is observed in the control participant, while a less defined and more variable pattern is evident in the FASD participant.

In the bottom panels, the running Fast Fourier Transform (FFT) shows the spectral representation of the response. Higher spectral energy is observed in the control participant compared to the FASD participant, particularly in the frequency range corresponding to the fundamental frequency and its harmonics.

**Figure 2:**
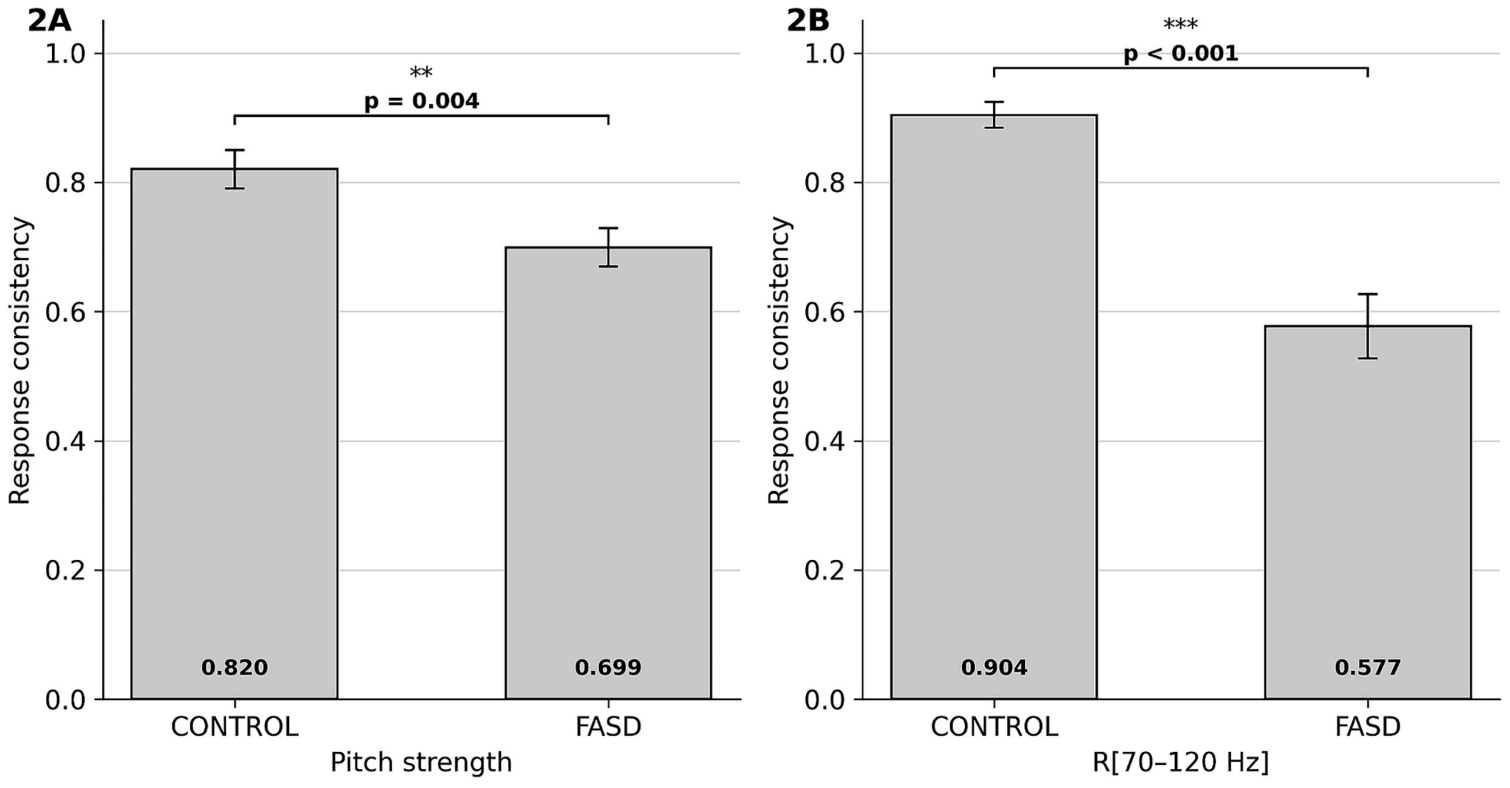
Consistency of FFR pitch tracking in control and FASD groups. **(A)** Consistency of F0 tracking of the FFR, evaluated using the Brainstem Toolbox pitch-tracking algorithm (*Analysis 1*), in control and FASD groups. Bars represent mean values and error bars indicate the standard error of the mean. The FASD group showed significantly lower response consistency compared to the control group (*p* = 0.004). **(B)** Stimulus–response correlation of the FFR within the F0 range (70–120 Hz), obtained using spectral analysis (*Analysis 2*), in control and FASD groups. Bars represent mean values and error bars indicate the standard error of the mean. The FASD group showed significantly lower correlation values compared to the control group (*p* < 0.001).

The consistency of F0 tracking was evaluated using two complementary analytical approaches (Figure 2).

First, pitch tracking consistency was assessed using the Brainstem Toolbox algorithm, which estimates the correlation between the stimulus and the neural response across the analyzed time window (Figure 2A). The FASD group showed significantly lower consistency values (mean = 0.699) compared to the control group (mean = 0.820; *p* = 0.004) (Supplementary Table S2).

Second, stimulus–response correlation was analyzed within the spectral range corresponding to the fundamental frequency (70–120 Hz) using the SmartEP® analysis module (Figure 2B). This analysis revealed more pronounced differences between groups, with lower correlation values in the FASD group (mean = 0.577) compared to the control group (mean = 0.904; Mann–Whitney U = 41.0, *p* < 0.001, rank-biserial *r* = 0.823), corresponding to a large effect size.

**Figure 3:**
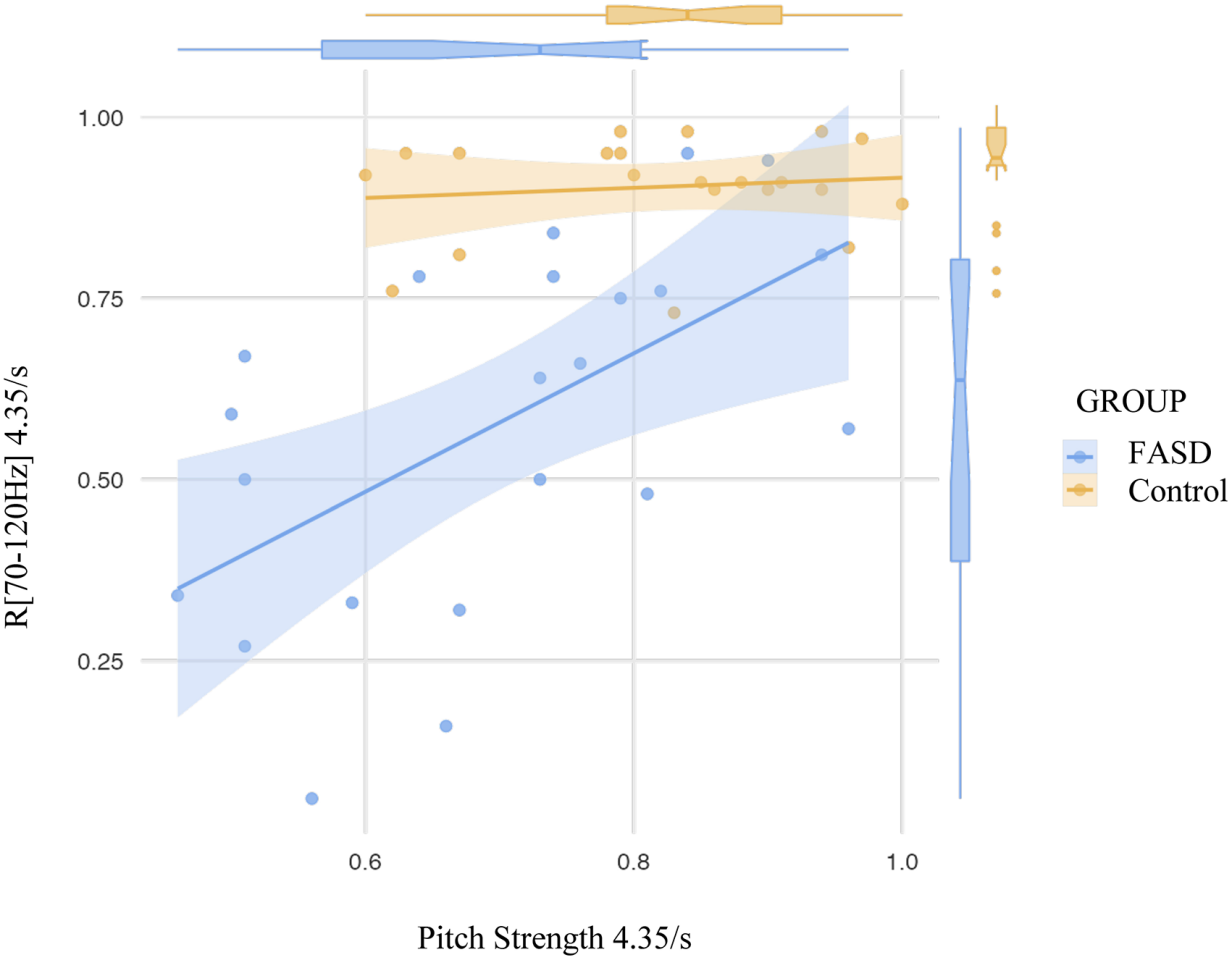
Relationship between two measures of FFR pitch tracking and interindividual variability in control and FASD groups. Relationship between two measures of F0 tracking obtained from FFR analysis: stimulus–response correlation within the 70–120 Hz frequency range calculated using SmartEP® (*vertical axis*) and pitch strength obtained using the Brainstem Toolbox (Northwestern University, USA) (*horizontal axis*). Both measures were derived from the same electrophysiological recordings acquired at a stimulation rate of 4.35/s. Each point represents an individual subject, color-coded by group. Regression lines and their confidence intervals illustrate the relationship between both measures within each group. Statistical association was assessed using Spearman rank correlation. Marginal distributions depict the distribution and interindividual variability of each variable.

The relationship between pitch strength and stimulus–response correlation in the 70–120 Hz range was examined separately for each group (Figure 3). A significant positive correlation was observed in the FASD group (Spearman *ρ* = 0.57, *p* = 0.006), indicating that individuals with lower pitch tracking consistency also showed lower stimulus–response correlation. In the control group, no significant correlation was observed between the two measures (Spearman *ρ* = −0.08, *p* = 0.720), likely reflecting a ceiling effect given the high and homogeneous values observed in this group for both metrics.

Control participants showed values concentrated in higher ranges for both metrics, whereas the FASD group exhibited greater dispersion and overall lower values.

**Figure 4:**
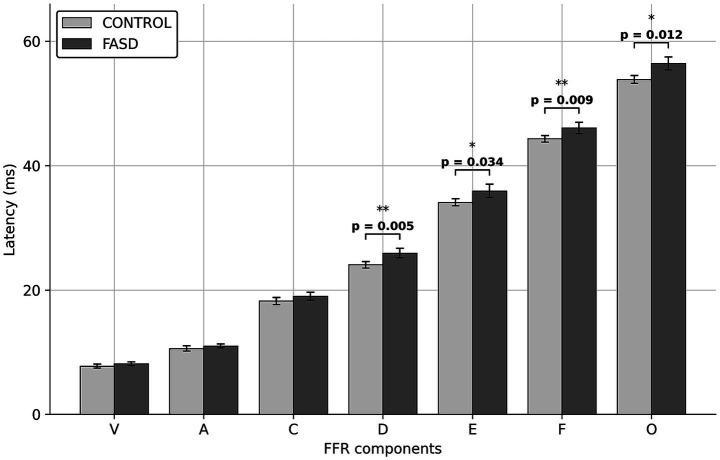
Mean latencies (± standard error) of FFR components (V–O complex) in control and FASD groups. Mean latencies (*± standard error*) of FFR components (V, A, C, D, E, F, and O) in control and FASD groups. Bars represent mean values and error bars indicate the standard error of the mean. Significant between-group differences were observed in components D (*p* = 0.005), E (*p* = 0.034), F (*p* = 0.009), and O (*p* = 0.012), with longer latencies in the FASD group compared to the control group. Statistical analysis was performed using non-parametric tests.

Latencies of the FFR V–O complex components were analyzed in both groups (Figure 4; Supplementary Table S1). Latencies were consistently longer in the FASD group compared to the control group, showing a progressive increase in group differences across the temporal sequence of the response.

Significant between-group differences were observed in components D (*p* = 0.005), E (*p* = 0.034), F (*p* = 0.009), and O (*p* = 0.012). Earlier components (V, A, and C) did not reach statistical significance, although a trend toward longer latencies in the FASD group was observed for components V (*p* = 0.054) and A (*p* = 0.087).

Additional analyses of wave V amplitude and signal-to-noise ratio (SNR) are presented in the Supplementary Figures S1, S2 and Supplementary Table S2. The FASD group showed lower mean wave V amplitude (mean = 0.305) compared to the control group (mean = 0.376), although this difference did not reach statistical significance (*p* = 0.471).

Similarly, SNR values measured within the 20–140 ms time window were lower in the FASD group (mean = 0.835) compared to the control group (mean = 0.915), showing a trend toward statistical significance (*p* = 0.081).

## 4 Discussion

The findings of the present study indicate alterations in the temporal precision of neural speech encoding in individuals with FASD, as reflected by convergent changes across multiple FFR measures. Specifically, individuals with FASD exhibited prolonged response latencies, reduced pitch-tracking consistency, lower SNR, and a non-significant tendency toward reduced wave V amplitude. Taken together, these findings suggest reduced precision and stability in neural speech encoding in individuals with FASD.

The FFR profile observed in the present study shares features with, but is also distinguishable from, patterns described in other neurodevelopmental conditions. In autism Spectrum disorder (ASD), Otto-Meyer et al. (2018) reported reduced trial-to-trial neural stability in speech-evoked FFRs, reflecting a failure to generate consistent responses across repetitions rather than a systematic reduction in response magnitude or timing precision. In contrast, the pattern observed in the present FASD sample is characterized by prolonged response latencies across multiple V–O complex components and reduced fidelity of fundamental frequency encoding — a profile more consistent with altered temporal precision than with neural instability per se. This distinction suggests that, despite both conditions involving auditory processing difficulties and neurodevelopmental origins, the underlying neural mechanisms may differ and could potentially be differentiated using FFR measures.

A closer parallel may be found with the FFR profile reported in neonates born after fetal growth restriction (FGR), another condition involving prenatal insult that passes standard audiological screening undetected. Ribas-Prats et al. (2022) reported attenuated signal-to-noise ratio in the vowel region of the speech-evoked FFR in FGR neonates, reflecting deficient neural encoding of the fundamental frequency of speech — a finding broadly consistent with the reduced pitch tracking consistency and lower stimulus–response correlation observed in the present FASD sample. This convergence suggests that prenatal adversity, regardless of its specific etiology, may leave a detectable electrophysiological signature at the level of subcortical auditory processing, and supports the potential of FFR as a sensitive biomarker of neural encoding deficits in populations with prenatal risk factors.

To our knowledge, this is the first study to characterize neural speech encoding in FASD using FFR. As such, the present work represents an initial step toward the use of objective electrophysiological measures in this population.

The findings reported here are consistent with previous studies using event-related potentials (ERPs), which have described electrophysiological alterations in components such as P2, N2, and P3. These results support the notion that prenatal alcohol exposure may affect multiple stages of auditory processing. In addition, studies using objective EEG and quantitative EEG (QEEG) measures have reported alterations in cognitive function in individuals with FASD, further supporting the presence of widespread neurophysiological changes in this population (Gerstner et al., 2024).

Unlike Auditory Brainstem Response (ABR), which is traditionally used to assess the integrity and synchrony of the early auditory pathway, and Cortical Auditory Evoked Potentials (CAEPs), which reflect higher-level stages of auditory processing, FFR provides a more detailed characterization of the temporal and spectral encoding of complex sounds.

This encoding has traditionally been associated with subcortical auditory structures, particularly within the midbrain, which play a key role in the integration and representation of acoustic information. However, FFR is increasingly understood as reflecting the integrated activity of multiple neural sources across the auditory system, including cortical contributions and top-down modulation depending on stimulus characteristics (Schochat et al., 2017; Bidelman, 2018; Gorina-Careta et al., 2021).

In clinical practice, the diagnosis of APD is generally based on the convergence of behavioral and electrophysiological measures (Brown and Musiek, 2013; Musiek and Chermak, 2015). However, the reliability of behavioral measures may be influenced by individual patient characteristics, and their interpretation depends on adequate cooperation. This is not always feasible in individuals with FASD, due to associated attentional and behavioral difficulties (Felgueras et al., 2024). In this context, FFR offers a clear advantage, as it does not require active participation from the subject and provides an objective measure of neural speech encoding. This makes FFR particularly suitable for assessing auditory function in populations with limited cooperation.

Moreover, its sensitivity to detect dysfunction at early stages of auditory processing suggests that FFR may help identify alterations that are not evident in conventional audiological evaluation. This characteristic also supports its potential use in guiding targeted interventions — including auditory training programs targeting speech encoding, language and phonological intervention, and psychoeducational support addressing listening and attentional difficulties — and in monitoring the effectiveness of such therapeutic programs in individuals with FASD (Skoe and Kraus, 2024; Skoe and Kraus, 2010).

Consistent with this, previous studies have demonstrated that brainstem responses to speech stimuli closely reflect the acoustic properties of the signal, providing access to the neural encoding of sound at early stages of auditory processing (Johnson et al., 2005).

The findings of the present study highlight the need for further research in this area, including larger sample sizes, the development of standardized protocols, and the integration of behavioral and electrophysiological measures, in order to advance toward a more precise characterization of auditory function in this population.

Although the results support the value of FFR in the study of auditory processing, further work is required to establish normative data, explore its applicability across different clinical populations, and facilitate its implementation in clinical practice.

Importantly, this study does not aim to establish a direct etiological link between prenatal alcohol exposure (PAE) and auditory processing alterations. The observed findings may not be exclusively attributable to alcohol exposure, as other prenatal factors could have contributed to the neurodevelopmental profile of these individuals. Therefore, no causal relationship is proposed, and the descriptive, quasi-experimental design of the study limits the interpretation of the results.

Given the ethical and practical constraints of studying prenatal alcohol exposure in humans, an alternative approach is to investigate individuals already diagnosed with FASD. In parallel, animal models have provided valuable insights into the teratogenic effects of PAE, including structural, neuronal, and functional alterations, as well as auditory deficits in exposed models (Akison et al., 2024; Lipinski et al., 2012; Jessup et al., 2025; Krawczyk et al., 2016).

## 5 Limitations

Several limitations of the present study must be acknowledged. First, the two groups differed significantly in age (mean difference ≈ 9.6 years; Mann–Whitney U = 25.5, *p* = 0.005, *r* = 0.67). Given the well-documented sensitivity of the FFR to developmental maturation — including systematic changes in response latency, pitch-tracking strength, and amplitude across the lifespan (Skoe and Kraus, 2010; Krizman and Kraus, 2019)— it cannot be excluded that some of the observed group differences reflect normal maturational variation rather than, or in addition to, FASD-related neural alterations. Recruitment of individuals with a confirmed FASD diagnosis was conducted through a specialized association of adoptive families, representing the only viable access pathway to this clinically underserved population. This constraint precluded strict age matching and underscores the need for future studies with age-matched designs and larger samples, for which the present work provides the methodological groundwork.

Second, sex was not controlled for in the statistical analyses. Research has consistently demonstrated sex-related differences in subcortical auditory processing, including FFR latency, amplitude, and pitch-tracking fidelity (Krizman et al., 2019). Future studies should include sex as a covariate in statistical models.

Third, formal assessment of language background was not conducted. While all participants were native Spanish speakers, potential Spanish-English bilingualism in the subgroup of control participants recruited in Miami cannot be excluded. Given the established sensitivity of the FFR to linguistic experience (Krizman et al., 2012), this represents an additional variable to be controlled in future research.

Fourth, the ear was used as the unit of analysis, which introduces potential non-independence between ears from the same participant. No laterality analysis comparing right vs. left ear responses was performed, as this was outside the scope of the present exploratory study and the sample was not powered for such comparisons. Future studies with larger samples should consider mixed-effects models accounting for the nested data structure.

Fifth, FFR component latencies were identified manually by a single trained evaluator, without inter-rater reliability assessment. Future studies should incorporate independent blind identification by a second evaluator.

Sixth, more than 10 independent statistical comparisons were performed without correction for multiple testing. Within the exploratory framework of this study, this increases the probability of false positive findings among results with *p*-values close to the significance threshold. The stimulus–response correlation in the F0 range (R[70–120 Hz]: *p* < 0.001, rank-biserial *r* = 0.823) constitutes the most robust finding of the study.

In light of these limitations, the present study is explicitly framed as an exploratory proof-of-concept investigation. The observed group differences cannot be specifically attributed to FASD, and the findings should be interpreted as hypothesis-generating rather than confirmatory. This first application of FFR in FASD nevertheless establishes the feasibility of the methodology in this population and provides the empirical foundation for future controlled research.

## 6 Conclusion

This exploratory proof-of-concept study provides preliminary evidence of altered neural speech encoding in individuals with FASD, as reflected by reduced pitch-tracking consistency, prolonged response latencies, and decreased signal quality in FFR. Importantly, the most robust finding — reduced stimulus–response correlation in the fundamental frequency range (R[70–120 Hz]: *p* < 0.001, rank-biserial *r* = 0.823) — supports the reliability of the observed pattern within the exploratory framework of this study.

These alterations were observed despite normal peripheral hearing, suggesting that listening difficulties in this population may arise from reduced precision and stability in neural encoding rather than from deficits at the peripheral level. However, given the uncontrolled confounds identified — particularly the age difference between groups — the observed differences cannot be specifically attributed to FASD, and the findings should be interpreted as hypothesis-generating rather than confirmatory.

To our knowledge, this is the first application of FFR in individuals with FASD. As such, this work establishes the feasibility of the methodology in this population and provides the empirical foundation for future controlled research with age-matched designs, larger samples, and formal control of demographic variables. FFR represents a promising objective tool for the characterization of auditory processing in populations where behavioral assessment is limited, and future studies should explore its potential as a biomarker for monitoring intervention outcomes in FASD.

## Data Availability

The original contributions presented in the study are included in the article/Supplementary material, further inquiries can be directed to the corresponding author.
