# Supplementary material for: Neural speech encoding in fetal alcohol spectrum disorder: an exploratory study using frequency-following responses
Source: Front Neurosci. 2026 Jul 16;20:1871045. doi: 10.3389/fnins.2026.1871045 (PMC13422169; doi:10.3389/fnins.2026.1871045)
Supplement: Supplementary file 1 [file Supplementary_file_1.pdf]

Supplementary Material

1 Supplementary Figures and Tables

1.1 Supplementary Figures

Supplementary Figure S1. Mean (± standard error) wave V amplitude of the FFR in control and FASD groups

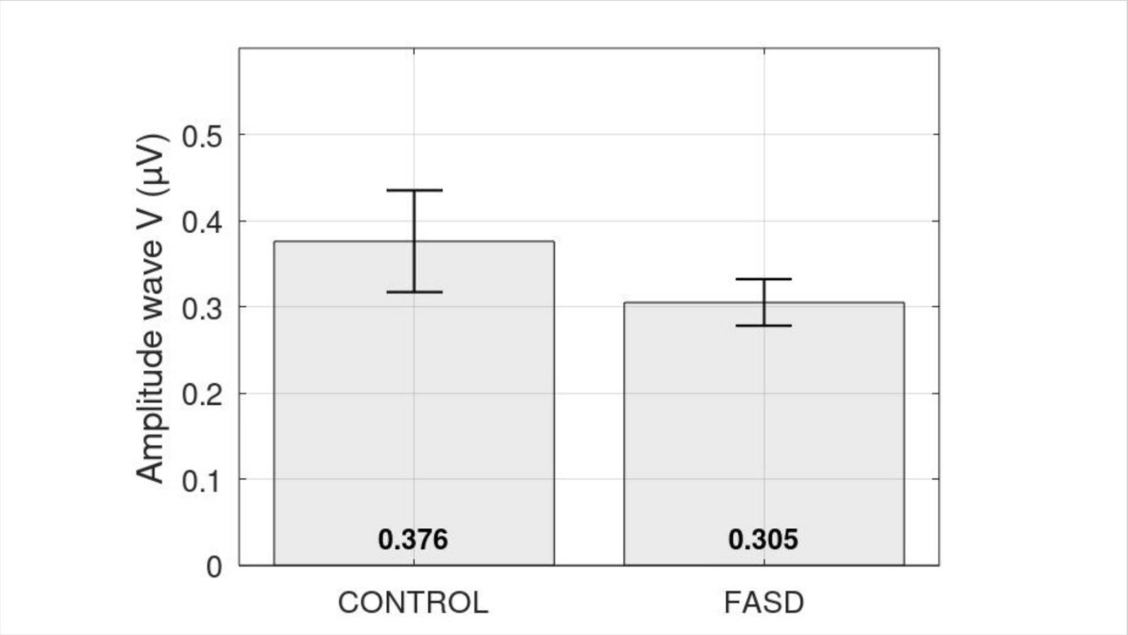

Supplementary Figure S1. The figure shows the mean (± standard error) amplitude of the V wave of the FFR in the control and FASD groups. A reduction in mean amplitude was observed in the FASD group (mean = 0.305 µV) compared to the control group (mean = 0.376 µV); however, this difference did not reach statistical significance (Mann–Whitney U test, $p = 0.470$). The standard error was 0.059 µV for the control group and 0.027 µV for the FASD group.

Supplementary Figure S2. SNR of the FFR in control and FASD groups (20–140 ms time window).

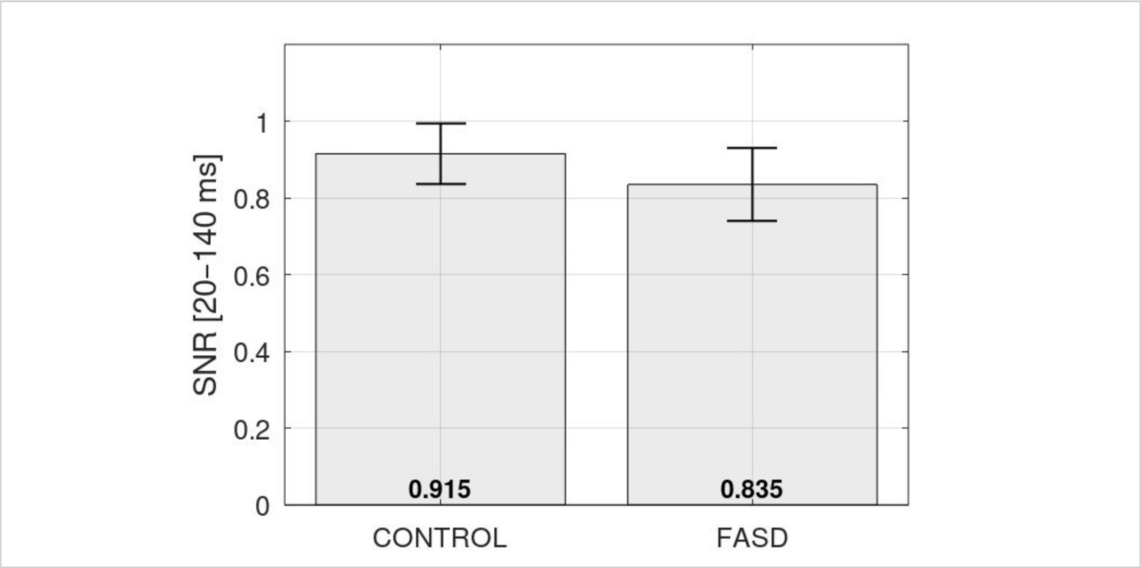

Supplementary Figure S2. The figure shows the mean SNR of the FFR in the 20–140 ms time window for the control and FASD groups. Bars represent mean values and error bars indicate the standard error of the mean. Lower SNR values were observed in the FASD group (mean = 0.835) compared to the control group (mean = 0.915). This difference showed a trend toward statistical significance but did not reach significance ($p = 0.081$).

1.2 Supplementary Tables

Supplementary Table S1. Descriptive statistics and inferential results for FFR V–O complex latencies in control and FASD groups.

	GROUP	N	Mean	SE	SD	IQR	Range	U	p	r
**V (ms)**	FASD	22	8.14	0.30	1.43	0.65	6.08	164.5	0.054	0.288
	control	21	7.75	0.32	1.48	0.98	7.13			
**A (ms)**	FASD	22	10.97	0.32	1.48	1.46	6.52	174.5	0.087	0.245
	control	21	10.59	0.43	1.99	1.65	9.22			
**C (ms)**	FASD	22	18.99	0.65	3.03	4.81	9.38	204.0	0.260	0.117
	control	21	18.22	0.57	2.63	2.92	9.89			
**D (ms)**	FASD	22	25.92	0.78	3.64	1.45	14.92	124.0	0.005	0.463
	control	21	24.05	0.53	2.43	2.10	10.58			
**E (ms)**	FASD	22	35.93	1.06	4.96	2.51	21.97	155.5	0.034	0.327
	control	21	34.09	0.58	2.64	2.45	14.17			
**F (ms)**	FASD	22	46.06	0.92	4.29	3.64	18.67	132.5	0.009	0.426
	control	21	44.33	0.52	2.40	1.42	12.07			
**O (ms)**	FASD	22	56.43	1.05	4.91	5.39	18.60	137.0	0.012	0.407
	control	21	53.86	0.62	2.84	2.22	12.25			

Supplementary Table S1. Latencies are expressed in milliseconds (ms). The table includes sample size (N), mean, standard error of the mean (SE), standard deviation (SD), interquartile range (IQR), total range, Mann-Whitney U statistic (U), exact p-value (p), and rank-biserial correlation as effect size (r). Statistical comparisons performed using non-parametric tests (Mann-Whitney U). U, p, and r values are reported in the FASD row for each component.

Supplementary Table S2. Descriptive statistics and inferential results for FFR pitch tracking, stimulus-response correlation, wave V amplitude, and signal-to-noise ratio in control and FASD groups.

	GROUP	N	Mean	SE	SD	IQR	Range	U	p	r
**Pitch Strength**	FASD	22	0.699	0.03	0.15	0.24	0.50	120.5	0.004	0.478
	Control	21	0.820	0.03	0.12	0.13	0.40			
**R[70–120 Hz]**	FASD	22	0.577	0.05	0.25	0.40	0.89	41.0	<0.001	0.823
	Control	21	0.904	0.02	0.07	0.05	0.25			
**Wave V (µV)**	FASD	22	0.305	0.03	0.13	0.14	0.61	227.5	0.471	0.015
	Control	21	0.376	0.06	0.27	0.29	0.95			
**SNR [20–140 MS]**	FASD	22	0.835	0.10	0.45	0.33	1.89	173.0	0.081	0.251
	Control	21	0.915	0.08	0.36	0.41	1.55			

Supplementary Table S2. The table includes sample size (N), mean, standard error of the mean (SE), standard deviation (SD), interquartile range (IQR), total range, Mann-Whitney U statistic (U), exact p-value (p), and rank-biserial correlation as effect size (r). Statistical comparisons performed using non-parametric tests (Mann-Whitney U). U, p, and r values are reported in the FASD row for each variable.
